# Green coffee extract modifies body weight, serum lipids and TNF-α in high-fat diet-induced obese rats

**DOI:** 10.1186/s13104-020-05052-y

**Published:** 2020-04-10

**Authors:** Cimi Ilmiawati, Fajar Fitri, Zelly Dia Rofinda, Mohamad Reza

**Affiliations:** 1 Department of Pharmacology, Faculty of Medicine, Andalas University, Main Campus at Limau Manis, Gedung A Lantai 1, Pauh, Padang, 25166 West Sumatra Indonesia; 2 Postgraduate Student, Graduate Program of Biomedical Science, Faculty of Medicine, Andalas University, Padang, West Sumatra Indonesia; 3 Department of Clinical Pathology, Faculty of Medicine, Andalas University, Padang, West Sumatra Indonesia; 4 Department of Biology, Faculty of Medicine, Andalas University, Padang, West Sumatra Indonesia

**Keywords:** Body weight, Cholesterol, Green coffee extract, Lipid profile, Obesity, TNF-α

## Abstract

**Objective:**

Currently, there are many efforts to find functional nutrients for obesity management, and the green coffee extract is a potential candidate. This study aimed to examine the effect of low dose administration of  green coffee extract on body weight, serum lipids, and TNF-α level in high-fat diet-induced obese rats.

**Results:**

Administration of green coffee extract to high-fat diet-induced obese male Wistar rats (*Rattus norvegicus*) reduced body weight, total serum cholesterol, and triglyceride at the dose of 10, 20, and 40 mg/kg BW/day; lowered serum LDL-cholesterol at the treatment dose of 20 mg/kg BW/day (p < 0.05). The effective dose to decrease serum TNF-α level was 40 mg/kg BW/day, while the effective dose to improve the lipid profile was 10 mg/kg BW/day. These results support the potential use of green coffee extract as a functional nutrient in the management of obesity.

## Introduction

Obesity is a universal public health problem characterized by increased adiposity, particularly in the abdominal region, which associated with increased cholesterol levels. Obesity often presents with dyslipidemia, where there is a high level of triglycerides (TGs), low-density lipoprotein (LDL)-cholesterol, and a low level of high-density lipoprotein (HDL)-cholesterol [1, 2]. As a chronic inflammatory condition, serum TNF-α level is elevated in obesity [3]. Obese rats have a 22% higher TNF-α level compare to normoweight rats [4]. Obese subjects are known to produce excess TNF-α in adipose and muscle tissues [5], where it plays a role in insulin resistance [6].

An epidemiological study showed that drinking coffee can have a weight-loss effect due to its chlorogenic acid (CGA) content [7]. Recently, there is an increase in a study examining the impact of green coffee extract (GCE) on obesity. GCE contains CGA, a phenolic compound with antioxidant property. CGA increases lipid metabolism, decreases triglyceride and cholesterol levels, and increases plasma adiponectin level [8]. Moreover, GCE has been shown to reduce visceral fat mass [9] significantly. In in vivo tudies in mice and rats,  CGA has been shown to regulate glucose and lipid metabolism, increased insulin sensitivity, and improved obesity [10]. Animal studies examining the effect of GCE on body weight, showed conflicting results [7, 11, 12]. Furthermore, the effect of GCE on serum lipid profile was previously seen in rodents administered relatively high doses of GCE or CGA [7, 12]. The objectives of the current study were to observe the impact of GCE on body weight, serum lipid profile, and serum TNF-α of obese rats by administrating dosing regimen lower than previously published studies.

## Main text

### Methods

#### Animals and diets

This study was approved by the Ethics Committee of the Faculty of Medicine Andalas University (No.381/KEP/FK/2017) and was conducted according to the institutional guidelines for animal research. Eight-week-old male Wistar rats (*Rattus norvegicus*) weighed around 200 g were obtained from an experimental animal breeding company (Tiput Abadi Jaya Peternakan Hewan Uji, Yogyakarta, Indonesia). They were acclimatized while fed with standard chow ad libitum. The rats were housed in a 25 °C room with 12 h light/dark cycle. After acclimatization, a group of male rats (n = 5) were randomly picked and assigned as the negative control group, which was fed standard chow ad libitum during the experiment, against which obesity induction was measured. A positive control group (n = 5) was fed standard chow (5% fat, 16% protein, crude fiber 8%, ash content 10%, water content 12%) and cheddar cheese ad libitum (high-fat diet (HFD)) throughout the experiment. Other groups of rats (the treatment groups, n = 15) were fed with standard chow supplemented with cheddar cheese to induce obesity for eight weeks. The cheddar cheese contained 33.1% fat, 25% protein, 1.3% carbohydrate (wt/wt), and energy 4 kcal/g. The cheese was given along with the standard chow in the same container, and the rats had free access to their diet. We did not observe a preference for one of the diets. Obesity induction was considered successful when there is a significant weight gain compared to the standard diet group (Additional file 1: Figure S1). Treatment groups were separated into three weight-matched groups (each n = 5) and were given a HFD and GCE at 10, 20, and 40 mg/kg body weight (BW)/day, respectively, for 13 days [7]. The GCE doses were lower than used in previous studies [7, 12]. The outcomes of the treatment were body weight, serum lipids levels, and serum TNF- α level.

#### GCE

GCE used in this study was the commercial product of *Hendel Exitox Green Coffee Bean*^*®*^ (Jakarta, Indonesia). Each capsule of this product contains 500 mg of GCE with 20.5–56.5 mg of CGA. The GCE was administered orally by gavage at the experimental dose of 10, 20, and 40 mg/kg BW per day.

#### Serum lipids and TNF-α analysis

After 13 days of treatment with GCE, on day 14, rats were anesthetized with diethyl ether at a concentration of 1.9%, and blood was drawn from the orbital sinus for measurement of serum lipids (total cholesterol, triglycerides, LDL-cholesterol, HDL-cholesterol) and TNF-α. Blood samples were transferred into a tube and were centrifuged to separate the sera. Sera were analyzed at the Laboratory of Biochemistry Faculty of Medicine Andalas University according to standard methods. TNF-α in serum samples were analyzed at the Laboratory of Biomedicine Faculty of Medicine Andalas University by using ELISA kit (Rat TNF-α ELISA Kit; Elabscience). Rats were sacrificed by cervical dislocation.

#### Statistical analysis

Data were checked for normal distribution by Shapiro–Wilk test. Differences in groups’ means were analyzed by *One Way ANOVA,* followed by Bonferroni’s *post hoc* test. Data were considered statistically significant when p-value < 0.05.

### Results

All animals (n = 25) were healthy throughout the experiment. First, we assessed the success of HFD-induced obesity by comparing rats fed standard chow (n = 5; negative control) with those fed standard chow plus cheese (n = 20; induced obesity group). Afterward, the induced obesity group was randomly separated into four groups (each n = 5) and subjected to GCE treatments and further analysis.

#### The effect of GCE on body weight of HFD-induced obese rats

**Fig. 1:**
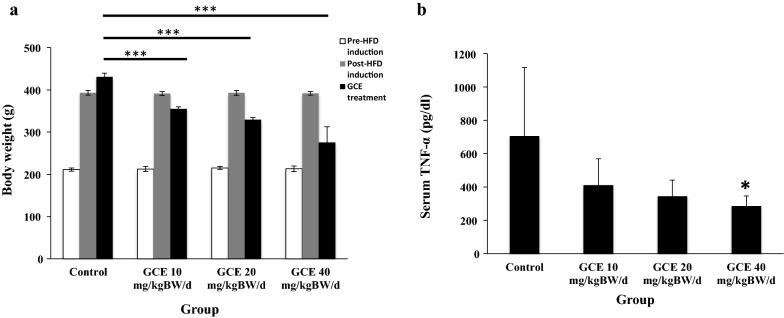
**a** The weight of male rats after a high fat diet (HFD) induction followed by 13 days of green coffee extract (GCE) treatment. The control group was fed HFD and placebo; treatment groups were fed HFD + GCE at 10, 20, and 40 mg/kg BW/day, respectively. ***p <0.001 (Bonferroni test compared to control group). **b** Serum TNF-α levels (pg/dl) in male rats after a high fat diet (HFD) induction followed by 13 days of green coffee extract (GCE) treatment. The control group was fed HFD and placebo; treatment groups were fed HFD + GCE at 10, 20, and 40 mg/kg BW/day, respectively. *p <0.05 (Bonferroni test compared to control group)

Animals fed HFD for eight weeks; all showed significantly increased body weight (Fig. 1a). Treatment with GCE for various doses (10, 20, and 40 mg/kg BW/day) for 13 days resulted in a statistically significant weight loss compared to the control group (Bonferroni test; p < 0.001) in a dose-dependent manner (Fig. 1a).

#### The effect of GCE on serum TNF-α of HFD-induced obese rats

GCE treatment for 13 days on HFD-induced obese male rats lowered TNF-α at the dose of 40 mg/kg BW/day, as shown in Fig. 1b (Bonferroni test; p < 0.05).

#### The effect of GCE on serum lipids of HFD-induced obese rats

**Fig. 2:**
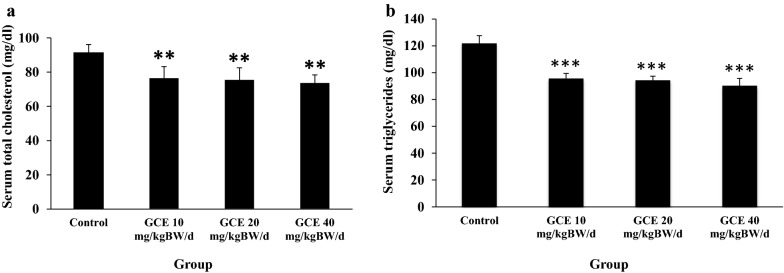
**a** Serum total cholesterol levels (mg/dl) in male rats after a high fat diet (HFD) induction followed by 13 days of green coffee extract (GCE) treatment. The control group was fed HFD and placebo; treatment groups were fed HFD + GCE at 10, 20, and 40 mg/kg BW/day, respectively. **p <0.01; ***p <0.001 (Bonferroni test compared to control group). **b** Serum triglycerides levels (mg/dl) in male rats after a high fat diet (HFD) induction followed by 13 d of green coffee extract (GCE) treatment. The control group was fed HFD and placebo; treatment groups were fed HFD + GCE at 10, 20, and 40 mg/kg BW/day, respectively. ***p <0.001 (Bonferroni test compared to control group)

**Fig. 3:**
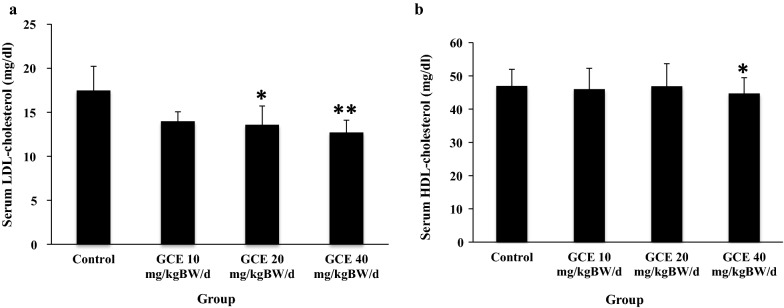
**a** Serum LDL-cholesterol levels (mg/dl) in male rats after a high fat diet (HFD) induction followed by 13 days of green coffee extract (GCE) treatment. The control group was fed HFD and placebo; treatment groups were fed HFD + GCE at 10, 20, and 40 mg/kg BW/day, respectively. *p <0.05;**p <0.01 (Bonferroni test compared to control group). **b** Serum HDL-cholesterol levels (mg/dl) in male rats after a high fat diet (HFD) induction followed by 13 days of green coffee extract (GCE) treatment. The control group was fed HFD and placebo; treatment groups were fed HFD + GCE at 10, 20, and 40 mg/kgBW/day, respectively. *p <0.05 (Bonferroni test compared to control group)

Obese male rats treated with GCE at 10, 20, and 40 mg/kg BW/day for 13 days showed statistically significantly lower serum total cholesterol and triglycerides levels compared to the control group (Fig. 2a, b, respectively). Treatment with GCE at the dose of 20 and 40 mg/kg BW/day also resulted in statistically significantly lower serum LDL-cholesterol levels (Fig. 3a). GCE treatment showed no effect on serum HDL-cholesterol levels except at the dose of 40 mg/kg BW/day, where HDL-cholesterol level decreased slightly (Bonferroni test; p < 0.05) (Fig. 3b).

### Discussion

#### The effect of GCE on body weight

This study shows that HFD induces obesity in male rats, and the administration of low dose GCE resulted in weight loss in these obese rats. The CGA content in GCE has been shown to have an anti-obesity effect [7, 8, 13]. Previously, a study in mice given high-fat diet and GCE at 100–200 mg/kg BW showed that GCE supplementation decreased body weight gain [14]. The observed anti-obesity effect may work by suppressing lipogenesis and stimulating lipolysis [14].

Our results are in line with previous findings in mice that showed CGA affects obesity by lowering body fat accumulation through adipogenesis regulation [12]. CGA increases lipid metabolism in HFD-induced obese rats [8, 13]. GCE significantly reduced visceral fat accumulation, improved insulin resistance [9], and, when combined with energy-restricted diet, may lead to a significant reduction in body mass index, fat mass, and waist-hip ratio [15].

#### The effect of GCE on lipid profile

Antioxidant-rich foods known to lower serum cholesterol, LDL, and triglyceride levels. In this study, we found that low dose GCE administration lowers total serum cholesterol, triglycerides, and LDL-cholecsterol levels. GCE contains CGA, a potent antioxidant compound. It has been shown that CGA in green coffee is an active compound capable of increasing metabolism rate [17], increasing fatty acid oxidation [8, 17], and decreasing hepatic triglyceride [7] and total cholesterol levels [16]. Apart from the CGA, the polyphenols in coffee also had a property in lowering visceral fat accumulation [18]. Unfortunately, we did not perform abdominal dissection to quantify visceral fat in this study.

In the current study, we found that the GCE has a negative effect on serum HDL levels. Serum HDL was lower in groups receiving 40 mg/kg BW/day dose. In contrast, those receiving lower dose (10 and 20 mg/kg BW/day) showed higher HDL levels despite not being statistically significant. This result is in line with a previous study were 28 days of CGA intake lowered HDL level in male rats through regulation of hepatic PPAR-α expression [17, 19]. These results might be explained by the possibility that CGA works specifically through the pro-atherogenic pathway of cholesterol metabolism. A clinical study in obese women aged 20–45 years showed that GCE combined with calorie-restricted diet affected lipid metabolism through significant change in serum total cholesterol, LDL, and free fatty acid [15].

#### The effect of GCE on TNF-α

Our result showed that GCE decreased serum TNF-α statistically significantly in the group receiving 40 mg/kg BW/day dose. We showed that the effect on TNF-α is dose-dependent; however, the normal level of TNF-α (10–100 pg/ml) could not be attained with the given doses. A higher dose or more prolonged intake of GCE may result in a further decrease of TNF-α. It has been shown that CGA, the powerful antioxidant in GCE, attenuates serum levels of TNF-α in a liver inflammation model [20]. CGA may downregulate the activation of NF-ĸB, which leads to a lower level of ROS that inhibits the production of the pro-inflammatory cytokine, like TNF-α [21].

## Conclusion

Our study confirmed that low dose GCE has a beneficial effect on body weight and lowers total serum cholesterol, triglyceride, LDL, and TNF-α levels in high-fat diet-induced obese rats. Our findings strengthen the scientific evidence on the property of GCE in the management of obesity and hyperlipidemia.

## Limitations

- Abdominal fat was not weighed for obesity assessment
- The number of animals in each treatment group was small

## Supplementary information

**Additional file 1: Figure S1.** Changes in rats’ body weight (g) during the high-fat diet (HFD) induction and green coffee extract (GCE) treatment. Control (-) group ate a standard diet and served as a control against which the success of HFD diet was measured. Control (+) group received HFD diet and placebo. ***p<0.001 [Bonferroni test compared to Control (-)].

## Data Availability

Data from this study are available from CI on a reasonable request.

## Abbreviations

ANOVA: Analysis of variance
BW: Body weight
CGA: Chlorogenic acid
ELISA: Enzyme-linked immunosorbent assay
GCE: Green coffee extract
HDL: High-density lipoprotein
HFD: High-fat diet
LDL: Low-density lipoprotein
NF-κB: Nuclear factor-kappa B
PPAR-α: Peroxisome proliferator-activated receptor alpha
ROS: Reactive oxygen species
TNF-α: Tumor necrosis factor-alpha
