# Supplementary material for: Green coffee extract modifies body weight, serum lipids and TNF-α in high-fat diet-induced obese rats
Source: BMC Res Notes. 2020 Apr 10;13:208. doi: 10.1186/s13104-020-05052-y (PMC7149906; doi:10.1186/s13104-020-05052-y)
Supplement: Supplementary file 1 — Additional file 1: Figure S1. Changes in rats’ body weight (g) during the high-fat diet (HFD) induction and green coffee extract (GCE) treatment. Control (-) group ate a standard diet and served as a control against which the success of HFD diet was measured. Control (+) group received HFD diet and placebo. ***p<0.001 [Bonferroni test compared to Control (-)]. [file 13104_2020_5052_MOESM1_ESM.pptx]

## Slide 1
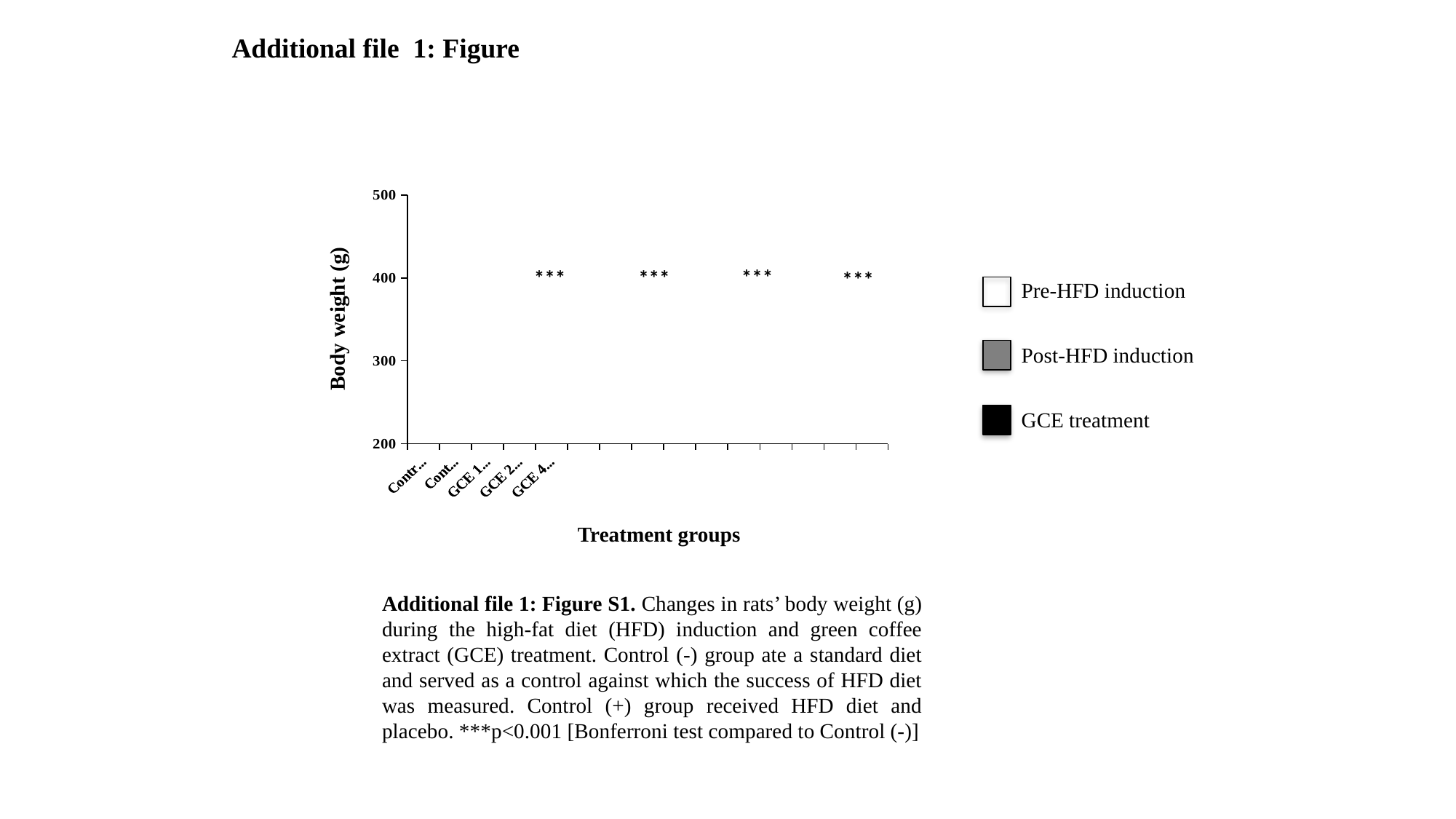

Additional file 1: Figure
### Chart
| Category | | | |
|---|---|---|---|
| Control (-) | 210.2 | 234.8 | 255.6 |
| Control (+) | 211.4 | 392.8 | 431.2 |
| GCE 10 mg/kg BW/d | 212.8 | 391.4 | 355.0 |
| GCE 20 mg/kgBW/d | 215.2 | 392.6 | 329.4 |
| GCE 40 mg/kgBW/d | 213.6 | 391.6 | 275.2 |***
***
***
***
Pre-HFD induction
Post-HFD induction
GCE treatment
Body weight (g)
Treatment groups
Additional file 1: Figure S1. Changes in rats’ body weight (g) during the high-fat diet (HFD) induction and green coffee extract (GCE) treatment. Control (-) group ate a standard diet and served as a control against which the success of HFD diet was measured. Control (+) group received HFD diet and placebo. ***p<0.001 [Bonferroni test compared to Control (-)]
